# The Gut Mycobiome Characterization of Gestational Diabetes Mellitus and Its Association With Dietary Intervention

**DOI:** 10.3389/fmicb.2022.892859

**Published:** 2022-06-15

**Authors:** Na Wu, Heng Mo, Qing Mu, Peng Liu, Guoli Liu, Weidong Yu

**Affiliations:** ^1^Department of Central Laboratory and Institute of Clinical Molecular Biology, Peking University People’s Hospital, Beijing, China; ^2^Department of Stomatology, Peking University People’s Hospital, Beijing, China; ^3^Department of Clinical Nutrition, Peking University People’s Hospital, Beijing, China; ^4^Department of Obstetrics and Gynecology, Peking University People’s Hospital, Beijing, China

**Keywords:** GDM, gut mycobiome, diet intervention, fungal-bacterial interaction, polysaccharide-producing genera

## Abstract

Gestational diabetes mellitus (GDM) is a high-risk pregnancy complication that is associated with metabolic disorder phenotypes, such as abnormal blood glucose and obesity. The active interface between gut microbiota and diet contributes to metabolic homeostasis in GDM. However, the contributions of gut mycobiome have been neglected. Here, we profiled the gut fungi between GDM and healthy subjects at two time points and investigate whether variations in gut mycobiome correlate with key features of host metabolism and diet management in this observational study. We identified that *Hanseniaspora, Torulaspora, Auricularia, Alternaria*, and *Candida* contributed to GDM patient clustering, indicating that these fungal taxa are associated with abnormal blood glucose levels, and the causality needs to be further explored. While *Penicillium, Ganoderma, Fusarium, Chaetomium*, and *Heterobasidion* had significant explanatory effects on healthy subject clustering. In addition, spearman analysis further indicated that blood glucose levels were negatively correlated with polysaccharide-producing genera, *Ganoderma*, which could be reshaped by the short-term diet. The *Penicillium* which was negatively correlates with metabolic parameters, also exhibited the antimicrobial attribute by the fungal-bacterial interaction analysis. These data suggest that host metabolic homeostasis in GDM may be influenced by variability in the mycobiome and could be reshaped by the diet intervention. This work reveals the potential significance of the gut mycobiome in health and has implications for the beneficial effects of diet intervention on host metabolic homeostasis through regulating gut fungal abundance and metabolites.

## Introduction

Alterations in the gut bacterial microbiota structure have been well studied in metabolic diseases such as gestational diabetes mellitus (GDM) (Wu et al., 2021), type 2 diabetes (Bielka et al., 2022) and obesity (Turnbaugh et al., 2009), and an increasing number of reports suggest that gut commensal fungi, as an important component of the intestinal microbiome, also influence host metabolic homeostasis (Shah et al., 2021; Zhang et al., 2021). Recent studies indicate that commensal fungi have the potential to influence host metabolism directly. In the context of obesity, fungal dysbiosis was observed, and the gut fungi at the phylum, family and genus levels were characterized by changes reflecting an abnormal composition (Mar Rodriguez et al., 2015). However, studies describing the role of the gut mycobiome in GDM patients remain scarce.

Gestational diabetes mellitus is one of the most common complications during pregnancy, affecting up to 25% of pregnant women worldwide (de Mendonca et al., 2022; Sparks et al., 2022). GDM patients are commonly treated by diet management to keep blood glucose levels within the normal range and to reduce the risk of GDM complications (Buchanan et al., 2012). Diet has an important function in the development of metabolic disease through its effects on the intestinal flora (Koren et al., 2012; Crusell et al., 2018; Wu et al., 2021). Few data from observational studies have revealed whether diet interventions affect the gut mycobiome in GDM patients. Our previous data suggest that diet is one of the important contributions to microbiota variation (Bassis, 2019; Johnson et al., 2019). However, an understanding of the differences in fungal populations between healthy pregnant women and individuals with GDM under routine dietary management remains unclear. To profile the composition of gut mycobiota in two groups of GDM and healthy subjects, and further investigate the association between the variations in gut mycobiome and key features of host metabolism under diet management, here, we profiled the gut fungal community structure of GDM patients and healthy subjects in the second trimester of pregnancy. We then performed a comparison of the fungal structures of GDM patients with routine dietary management to evaluate the role of short-term diet management on GDM patient gut microbiota. We then examined the links between gut fungi and clinical metabolic parameters. Our study supports the role of the gut mycobiome in GDM patient variation and suggests that diet management influences the GDM gut mycobiome, providing updated insight into the complex gut fungal ecology and its effects on host health.

## Materials and Methods

### Ethical Approval

This study was approved by the Conjoint Health Research Ethics Board of Peking University People’s Hospital, and informed consent forms were signed by all of the subjects prior to participation in this study. All experiments were performed in accordance with the approved guidelines and regulations.

### Patient Recruitment

The diagnosis of GDM patients was performed based on the diagnostic criteria recommended by the International Association of the Diabetes and Pregnancy Study Groups in 2011 as our previous study described (Lapolla et al., 2011; Wu et al., 2021). In brief, the threshold values of the Oral Glucose Tolerance Test (OGTT) were 5.1 at 0 h, 10.0 at 1 h, and 8.5 at 2 h. GDM patients were recruited based on the criteria. Healthy subjects were recruited based on matched pregnancy period. All subjects who met the following criteria were excluded: complicating diseases (such as known diabetes mellitus, hypertension, cardiovascular, pulmonary, autoimmune, joint, liver or kidney diseases; thyroid dysfunction; or any other disease), prebiotics/probiotics use, and antibiotic use during pregnancy.

### Diet Management and Stool Sample Collection

The initial treatment of GDM involves diet modification, glucose monitoring, and moderate exercise (Blumer et al., 2013; American Diabetes, 2014).

As our previous study described (Wu et al., 2021), stool samples of GDM patients and healthy pregnant women at 24–28 weeks of gestation were collected twice over a 2-week interval. The sample size was calculated by PASS software (NCSS LLC, Kaysville, UT, United States). With a sample size of 22 in each group should achieve more than 80% power to detect a difference between GDM patients with 0 h blood glucose level of 5.25 ± 1.49 (Mean ± SD) and healthy subjects with that of 4.29 ± 0.34 (Mean ± SD). In this study, stool samples from 23 GDM and 26 healthy subjects were included to reveal the gut fungal composition.

Gestational diabetes mellitus patients received diet intervention treatment during the 2 weeks. For healthy pregnant women, the second stool samples were collected at the end of 2 weeks without dietary management intervention. Stool samples from GDM and healthy subjects were collected at the time of enrollment for the first time. The second stool samples were collected at the end of the study after the 2-week. A flow chart illustrating the recruitment strategy of GDM and healthy subjects is shown in Supplementary Figure 1.

In summary, all the GDM participants received 2 weeks of dietary management and nutritional recommendations at enrollment, which showed the guidelines for the GDM subjects. Participants were instructed as adhering to follow the given dietary recommendations based on the wide consumption of cereals, legumes, skimmed dairy products, low-fat meat and fish, vegetables, fruit and no consumption of processed baked goods, fast foods, soft drinks, juices and alcohol (Wu et al., 2021). The low-glycemic, low-saturated fat diet had a target macronutrient composition of 35–45% carbohydrates (80% complex carbohydrates with a low glycemic index and 20% simple carbohydrates), 18–20% protein (50% animal and 50% vegetable) and 35% fat (16% mono-unsaturated, 10% polyunsaturated and 9% saturated) with moderately low saturated fat levels, fiber intake of at least 20–25 g/day. The daily recommended calories were divided into small frequent meals to avoid ketonuria and acidosis, which frequently occurs because of prolonged fasting. The qualitative-quantitative method of current quotation (3-day food record), from three consecutive days, including two business days and one holiday day, was used to assess the diet of patients during the study period. The nutritionist was in continuous contact with the enrolled GDM subjects, through weekly telephone contact, to record the daily diet and to remain updated regarding the nutritional condition of the subjects as the study progressed. Patients were instructed to self-monitor their blood glucose by finger-prick capillary blood glucose tests at least four times per day.

### DNA Extraction

DNA was extracted from stool samples using the QIAamp DNA Stool Mini kit protocol (Qiagen, Germany). During DNA extraction, four fecal samples from GDM patients and four fecal samples from healthy subjects were excluded, which changed the sample size from 27 to 23 in the GDM group and from 30 to 26 in the healthy group.

### Fungal ITS1 rDNA Amplification and Sequencing

After DNA extraction, fungal ITS genes were amplified using ITS1-specific barcoded primers, as mentioned in a previous report. Briefly, the ITS1 region was amplified using the forward primer (5′– CTTGGTCATTTAGAGGAAGTAA-3′) and reverse primer (5′-GCTGCGTTCTTCATCGATGC-3′). The ITS1-specific primers were linked with the barcode and linker primers. After the purification of PCR products of the ITS1 genes, paired-end sequencing (2 × 125 bp) was performed by an Illumina NovaSeq 6000 sequencer.

### Fungal ITS1 rDNA Data Analysis

The raw tags were performed to obtain the high-quality clean tags according to the QIIME (V1.9.1^1^) quality controlled process (Caporaso et al., 2010). The tags were then performed using UCHIME algorithm (UCHIME Algorithm^2^) to detect and remove chimera sequences (Edgar et al., 2011).

Operational taxonomic units (OTUs) were identified at the 97% similarity level. The representative sequences of OTUs were aligned with the UNITE ITS database (Abarenkov et al., 2010). The abundance profile of the samples was generated at the phylum, genus and species levels from OTUs.

The comparison of the fungal alpha-diversity was performed using the Chao1 richness index and Shannon’s diversity index. Beta-diversity analyses were calculated based on OTUs, and principal coordinate analysis (PCoA) was performed based on the unweighted UniFrac distance metric (Lozupone and Knight, 2005). Then, the Adonis test was performed to reveal the significance of fungal composition differences between the groups (the separation of clusters) (Steyn and Ellis, 2009). Based on OTUs, a Venn diagram was drawn for the analysis of group-specific fungal taxa using the R package “VennDiagram” (Chen and Boutros, 2011). To build a phylogeny tree, the top 100 fungal OTUs were further analyzed to visualize the differences between the GDM and non-GDM groups using FastTree Software (Price et al., 2009). Furthermore, RDA analysis was performed to identify the contributors to the fungal community using R package “vegan” (Sheik et al., 2012).

### Analysis of Microbial Interaction Patterns

To analyze the microbial-fungal interaction patterns present in the different groups, pairwise comparisons of bacterial and fungal abundances at the genus level were performed to determine correlations with clinical parameters using the Mantel tests in R package “vegan” (Zhang and Boos, 1997). The bacterial abundance data were used from our previous study (Wu et al., 2021). Only significant correlations (*P* < 0.05 after false discovery rate correlation) are displayed.

### Statistical Analysis

SPSS (Statistical Package for Social Sciences) 22.0 software (SPSS Inc., Chicago, IL, United States) was used to perform statistical analysis of the clinical data. The fungal comparisons of groups were performed using the Mann–Whitney test. The Spearman rank correlation coefficient method was used to evaluate the associations between clinical indices and gut microbiota. The differences in alpha diversity and beta diversity between groups were assessed using Student’s *t* test. *P* < 0.05 was considered significantly different.

## Results

### Characteristics of the Participants

A total of 23 GDM subjects and 26 healthy pregnant women at 24–28 weeks of gestation were recruited at Peking University People’s Hospital. Stool samples of GDM patients and healthy pregnant women were collected twice over a 2-week interval, to evaluate the gut fungal differences between GDM patients and healthy pregnant women.

Clinical data from 23 GDM patients and 26 healthy controls are shown in Table 1. The mean age of the subjects was 33.2 ± 3.3 years for the GDM patients and 31.2 ± 2.6 years for the healthy subjects. There were only 2 years differences in age between the two groups (*P* = 0.013). The pre-pregnancy BMI value of the GDM group was 24.3 ± 4.1, which was significantly higher than the value of 21.4 ± 2.8 for the healthy group (*P* = 0.01), and the same trend was observed for the BMI at enrollment (27.2 ± 4.3 vs. 25.0 ± 2.9, GDM vs. healthy, *P* = 0.052). The GDM group had a markedly higher systolic BP (SBP) value than the control group (mean 125.0 ± 11.5 vs. 115.6 ± 15.0, GDM vs. healthy, *P* = 0.015), and a higher diastolic BP (DBP) value was found in GDM women than in healthy women (mean 78.2 ± 9.1 vs. 72.8 ± 8.7, GDM vs. healthy, *P* = 0.034). In the OGTT test, the GDM group had higher values at 0, 1, and 2 h than those of the healthy group (all *P* < 0.001).

**TABLE 1:** The clinical characteristics of 23 GDM patients and 26 healthy subjects.

	GDM (Mean ± SD)	Healthy (Mean ± SD)	*P-value*
Number	23	26	
Age	33.2 ± 3.3	31.2 ± 2.6	0.013
Pre-prepregnancy BMI (kg/m^2^)	24.3 ± 4.1	21.4 ± 2.8	0.010
Enrollment BMI (kg/m^2^)	27.2 ± 4.3	25.0 ± 2.9	0.052
Systolic BP (mmHg)	125.0 ± 11.5	115.6 ± 15.0	0.015
Diastolic BP (mmHg)	78.2 ± 9.1	72.8 ± 8.8	0.034
**OGTT(mmol/L)**			
0 min	5.3 ± 1.5	4.3 ± 0.3	0.000
60 min	10.4 ± 1.5	7.3 ± 1.4	0.000
120 min	8.7 ± 1.4	6.4 ± 1.2	0.000

### Differences in Gut Mycobiome Between the Healthy and Gestational Diabetes Mellitus Groups

**FIGURE 1:**
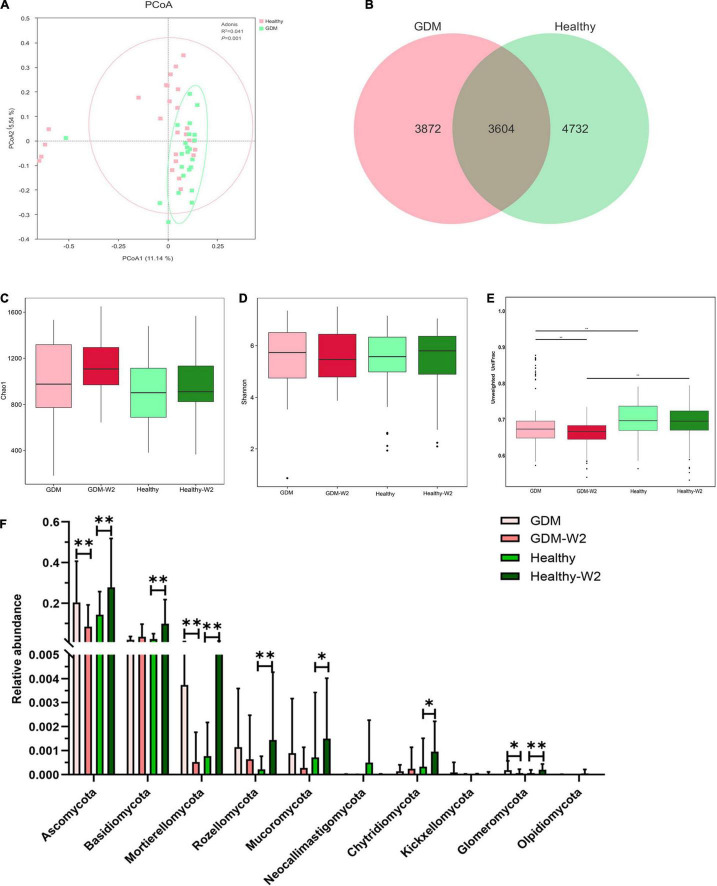
Comparison of the gut mycobiome composition between the GDM (*n* = 23) and healthy (*n* = 26) groups. **(A)** Principal coordinate analysis (PCoA) at the OTU level between the GDM and healthy groups at enrollment. **(B)** Venn diagram illustrating the overlap of the OTUs identified in the mycobiome between the GDM and healthy groups at enrollment. **(C)** Alpha-diversity based on the Chao 1 index at the OTU level. Mann-Whitney test. **(D)** Alpha-diversity based on the Shannon index at the OTU level. Mann-Whitney test. **(E)** Beta-diversity based on the unweighted UniFrac distance. Mann-Whitney test. **(F)** Comparison of the relative abundances at the phylum level among the four GDM and non-GDM groups. Mann-Whitney test, ^**^P < 0.01, *P < 0.05.

The gut mycobiome from 98 fecal samples from 23 GDM patients and 26 healthy subjects were profiled with fungal ITS gene sequencing. A total of 6,594,794 high-quality combined sequences (67,294 ± 6,113 sequences per sample) were ultimately produced, which corresponded to 14 known phyla, 366 families and 754 different genera. To demonstrate the differences in the gut mycobiome of the GDM patient and healthy subject groups, we explored the fungal composition of pregnant women with GDM and observed discrete clustering of intestinal mycobiome in the GDM patient and healthy subject groups at enrollment by PCoA analysis (*R*^2^ = 0.041, *P* = 0.001, Figure 1A). Additionally, to detect whether GDM had an effect on the gut microbiota, Venn diagrams were generated to assess the shared or unique OTUs in the GDM patient and control women at enrollment. The healthy group had more unique OTUs, with approximately 56.8% (4732/8336) unique OTUs compared with 51.8% (3872/7476) in GDM women, signifying that healthy pregnant women largely harbor unique inhabitant niches (Figure 1B).

The microbial alpha-diversity analysis was assessed by means of the Chao 1 and Shannon indices, and there was no difference between the healthy and GDM groups at enrollment and at the end of the study (Figures 1C,D). In addition, the beta-diversity was significantly increased in the healthy group at enrollment and at the end of the study (Figure 1E) relative to that of GDM subjects, suggesting increased fungal commensal diversity in the healthy group.

### The Fecal Mycobiome Composition Differs in Gestational Diabetes Mellitus Patients

We compared the relative abundance of fungi between the GDM and healthy groups at enrollment to further determine variations associated with the structure of the gut mycobiome in GDM patients. Ascomycota and Basidiomycota were the dominant phyla in the gut mycobiomes, and Ascomycota abundance was found to be 20.3% in the GDM group and 14.3% in the healthy group (Mann–Whitney test, *P* = 0.315) (Figure 1F). Basidiomycota abundance was 2.1% in the GDM group compared with 2.4% in the healthy group (Mann–Whitney test, *P* = 0.9). The relative abundances of the phyla Mortierellomycota (Mann–Whitney test, *P* = 0.054), Rozellomycota (Mann–Whitney test, *P* = 0.053) and Glomeromycota (Mann–Whitney test, *P* = 0.058) in the GDM patient mycobiome were higher than those of samples from healthy subjects. No significant differences were observed between the healthy subjects and the GDM subjects at enrollment for other phyla.

**FIGURE 2:**
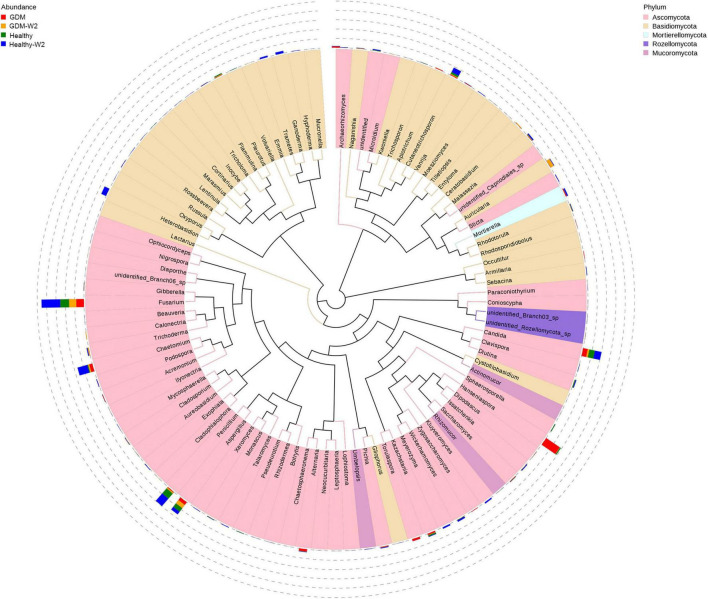
Phylogeny tree shown top 100 genera in the four groups, including the GDM and healthy and the GDM-W2 and healthy-W2 groups. 23 GDM patients and 26 healthy subjects.

Next, the top 100 genera were shown by phylogeny tree to compare the group differences at the genus level (Figure 2). Genera *Hanseniaspora*, *Torulaspora*, *Kazachstania*, *Trichoderma*, *Pichia*, *Acremonium*, *Sticta*, *Cladophialophora*, *Lophiostoma*, and *Gibberella*, belonging to Ascomycota, were the dominant genera in GDM subjects (Mann–Whitney test, all *P* value < 0.05) (Table 2). In contrast, four genera in the phyla Basidiomycota, *Ganoderma*, *Volvariella*, *Marasmius*, and *Tricholoma* and one genus *Rhizomucor* in the phylum Mucoromycota were significantly more abundant in the healthy subject mycobiome than in the GDM patient mycobiome. In addition, *Penicillium*, *Fusarium*, *Chaetomium* belonging to Ascomycota, and *Ganoderma*, *Heterobasidion*, *Cutaneotrichosporon*, and *Trametes* belonging to Basidiomycota appeared only in a subset of healthy subjects at the end of the study, whereas they were nearly absent in GDM subjects (Figure 2), suggesting the probiotic role of these fungal taxa.

**TABLE 2:** Genera that were different in the GDM and healthy groups.

Taxonomy	Phyla	*P* value	GDM mean	Healthy mean	GDM/Healthy
*Hanseniaspora*	Ascomycota	0	0.068	0.0035	Up
*Torulaspora*	Ascomycota	0.002	0.0085	0.00010	Up
*Kazachstania*	Ascomycota	0.028	0.0056	0.0050	Up
*Diutina*	Ascomycota	0.018	0.00052	0.0034	Down
*Trichoderma*	Ascomycota	0	0.00068	0.00050	Up
*Sphaerosporella*	Ascomycota	0.002	0	0.0031	Down
*Pichia*	Ascomycota	0.004	0.0026	0.00060	Up
*Pseudeurotium*	Ascomycota	0.009	5.7E-05	0.0016	Down
*unidentified*	Ascomycota	0	0.0050	0.00022	Up
*Acremonium*	Ascomycota	0.028	0.0016	0.00026	Up
*Sticta*	Ascomycota	0.024	0.00050	2.5E-06	Up
*Diaporthe*	Ascomycota	0.043	2.9E-06	3.2E-05	Down
*Cladophialophora*	Ascomycota	0.005	0.00012	2.9E-05	Up
*unidentified_Capnodiales*	Ascomycota	0.046	2.9E-06	7.8E-06	Down
*Chaetosphaeronema*	Ascomycota	0.028	0	0.00032	Down
*Lophiostoma*	Ascomycota	0.006	4.1E-05	1.6E-05	Up
*Gibberella*	Ascomycota	0.011	0.00078	0.00065	Up
*Ganoderma*	Basidiomycota	0.003	4.3E-05	0.00042	Down
*Ceratobasidium*	Basidiomycota	0	0.00063	1.7E-06	Up
*Malassezia*	Basidiomycota	0	0.0014	0.0013	Up
*Armillaria*	Basidiomycota	0.001	0.0016	8.4E-06	Up
*Volvariella*	Basidiomycota	0.001	0	0.0012	Down
*Marasmius*	Basidiomycota	0.007	1.9E-06	0.0010	Down
*Russula*	Basidiomycota	0.018	0.00050	0.00028	Up
*Tricholoma*	Basidiomycota	0.028	0	1.9E-05	Down
*Apiotrichum*	Basidiomycota	0	0.0022	1.9E-05	Up
*Vanrija*	Basidiomycota	0	0.0016	2.5E-06	Up
*Rhodosporidiobolus*	Basidiomycota	0	0.0010	6.6E-05	Up
*Occultifur*	Basidiomycota	0.017	0.00023	2.8E-05	Up
*Mortierella*	Mortierellomycota	0	0.0034	0.00069	Up
*Rhizomucor*	Mucoromycota	0.014	1.5E-05	0.00063	Down
*Umbelopsis*	Mucoromycota	0.013	0.00040	0	Up
*unidentified_Branch03*	Rozellomycota	0.024	0.00011	4.2E-06	Up
*unidentified_Rozellomycota*	Rozellomycota	0.046	0.00048	8.8E-05	Up

*Comparison of the mycobiota between the GDM patients and healthy subjects. Mean values are presented in two groups. The Mann–Whitney test was used to evaluate the two groups.*

In addition, the gut fungal differences of top 10 species between GDM and healthy subjects were addressed as Supplementary Figure 2. The levels of species *Hanseniaspora pseudoguilliermondii*, *Alternaria japonica*, and *Hanseniaspora meyeri* were significantly higher in GDM group than that in healthy group at enrollment. Meanwhile, *Penicillium chrysogenum* was remarkably enriched in gut mycobiota of healthy subjects, which was agreement with the genus findings.

### Key Factors Contributing to the Variation in Gestational Diabetes Mellitus and Healthy Subjects

**FIGURE 3:**
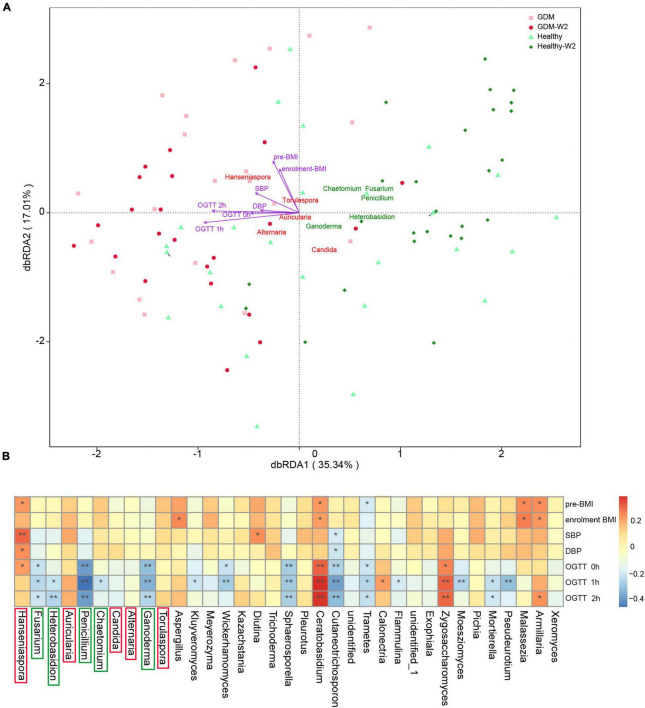
**(A)** Key contributors to fungal community variation was determined by RDA analysis, plotted on the two first RDA dimensions. **(B)** Heatmap analysis of the correlation between the gut mycobiome composition and clinical parameters, **P < 0.01, *P < 0.05.

To explore the factors contributing to the variation in the gut mycobiome of GDM and healthy subjects, RDA analysis was performed (Figure 3A). The variation in GDM and healthy subjects was explained by genus level differences in the gut mycobiome. Ten taxa were identified as the key contributors to gut fungal community variation. Among the ten taxa, *Hanseniaspora*, *Torulaspora*, *Auricularia*, *Alternaria*, and Candida contributed to GDM patient clustering, while *Penicillium*, *Ganoderma*, *Fusarium*, *Chaetomium*, and *Heterobasidion* had significant effects on healthy subject clustering, indicating that these fungal taxa are highly associated with abnormal blood glucose levels.

In addition, clinical metabolic parameters, including OGTT values (0, 1, and 2 h), BMI indices at enrollment, and blood pressure values (SBP and DBP), were evaluated for their association with fungal variation in the GDM and non-GDM groups. The gut fungal clustering varied among GDM and healthy participants and was associated with significance for host metabolism parameters by RDA analysis (Figure 3A and Supplementary Table 1). The 2-h OGTT value had the largest effect on the GDM status variation in the gut mycobiome (*R*^2^ = 0.47, *P* = 0.0005).

### Association Between Clinical Parameters and Fungal Taxa

Furthermore, Spearman correlation analysis was used to examine the association between fungal level and host factors (Figure 3B). The distribution of correlation coefficients by heatmap analysis showed that the *Zygosaccharomyces*, *Ceratobasidium*, *Armillaria*, *Calonectria*, and *Hanseniaspora* were positively correlated with OGTT values; among them, the *Zygosaccharomyces* and *Ceratobasidium* showed significant positive correlation with all the OGTT values (0, 1, and 2 h), indicating a disturbing effect on glucose usage. In contrast, the abundances of 14 fungal taxa were negatively correlated with the OGTT values, including *Penicillium*, *Ganoderma*, *Fusarium*, *Chaetomium*, *Heterobasidion*, *Kluyveromyces*, *Wickerhamomyces* and seven other genera, which is consistent with the findings shown in Figure 3A. Among the 14 genera, *Penicillium*, *Ganoderma*, *Fusarium*, *Chaetomium*, and *Heterobasidion* were indicated as important contributors for differentiating healthy subject samples from GDM patient samples.

Additionally, *Armillaria*, *Hanseniaspora*, *Aspergillus*, and *Ceratobasidium* abundances also showed positive correlations with BMI. *Hanseniaspora* and *Diutina* abundances showed positive correlations with GDM-correlated SBP/DBP measures. Among them, abundances of *Armillaria*, *Hanseniaspora*, and *Ceratobasidium* were also significantly higher in the GDM group than in the healthy group (Table 2).

### The Mycobiome Signature in Healthy Subjects at Two Time Points

**FIGURE 4:**
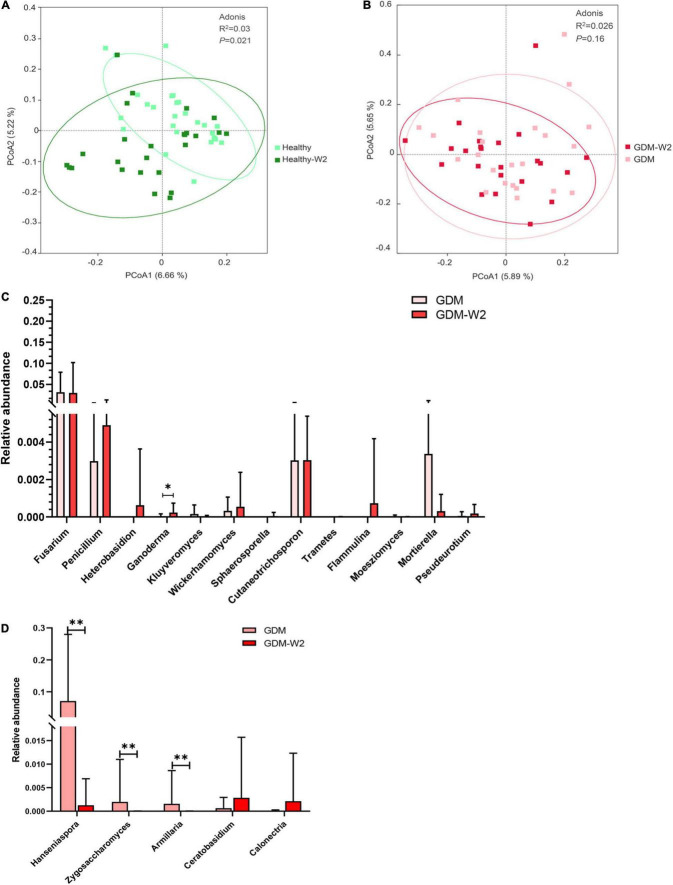
The microbial pattern between two time points. **(A)** Principal coordinate analysis (PCoA) at the OTU level between the healthy and healthy-W2 groups (*n* = 26). **(B)** Principal coordinate analysis (PCoA) at the OTU level between the GDM and GDM-W2 groups (*n* = 23). **(C)** Relative abundance of probiotic genera, in the GDM samples with or without diet intervention. Mann-Whitney test. **(D)** Relative abundance of genera positively related with metabolic scores, in the GDM samples with or without diet intervention. Mann-Whitney test, ^**^P < 0.01, *P < 0.05.

According to the PCoA plot (*R*^2^ = 0.03, *P* = 0.021), the healthy subjects indicated a remarkably different mycobiome pattern after 2 weeks (Figure 4A). The relative abundances of distinct fungal taxa were compared between healthy samples collected at enrollment and at the end of the study. At the phylum level, the mycobiome in Healthy-W2 samples showed significantly higher enrichment for fungal taxa, including the main phyla Ascomycota, Basidiomycota and five other phyla, than that in samples collected at enrollment (Figure 1F).

At the genus level, Healthy-W2 subjects developed a better fungal composition with increased *Penicillium*, *Mortierella*, *Cutaneotrichosporon*, *Wickerhamomyces*, *Chaetomium*, *Fusarium*, *Kluyveromyces*, *Heterobasidion*, *Trametes*, and *Ganoderma* (Supplementary Figure 3), which also represented a probiotic effect on the host blood glucose level (Figure 3).

### Mycobiome Signature After Dietary Intervention in Gestational Diabetes Mellitus Patients

The role of diet intervention on the phylogenetic structure of gut fungi during pregnancy remains underexplored in well-controlled models. To evaluate the role of dietary intervention on the GDM gut mycobiome, the fungal signature was profiled and compared between samples collected from GDM patients with and without dietary intervention. Based on the PCoA (*R*^2^ = 0.026, *P* = 0.16), there were no significant differences between GDM samples with and without diet management (Figure 4B). The fungal lineages were similar between all the cases at the two time points, which is in agreement with the bacterial findings shown in our previous study (Wu et al., 2021). The findings of bacterial and fungal composition revealed that the role of short-term diet management in GDM patient treatment is associated with the change in some specific bacterial and fungal taxa, rather than an alternative gut microbial pattern.

One more interesting observation is that GDM-W2 samples harbored more probiotic fungal genera driven by the 2 weeks of diet management than those of the samples at enrollment, including enriched *Ganoderma* (Figure 4C), which was negatively correlated with OGTT values and had a significant effect on healthy subject clustering (Figure 3B). In addition, three fungal genera (*Hanseniaspora*, *Zygosaccharomyces*, and *Armillaria*) correlated with higher blood glucose levels were absent in GDM-W2 samples obtained from patients after dietary intervention, compared to GDM samples at enrollment. Of particular interest, *Hanseniaspora*, the abundance of which increased in GDM groups, represented the key contributing factor to the separation of GDM patient from healthy subject samples (Figure 4D), suggesting that diet intervention could improve the fungal composition by decreasing GDM-related fungal taxa.

### Interaction Between Differential Fungal and Bacteria Genera

**FIGURE 5:**
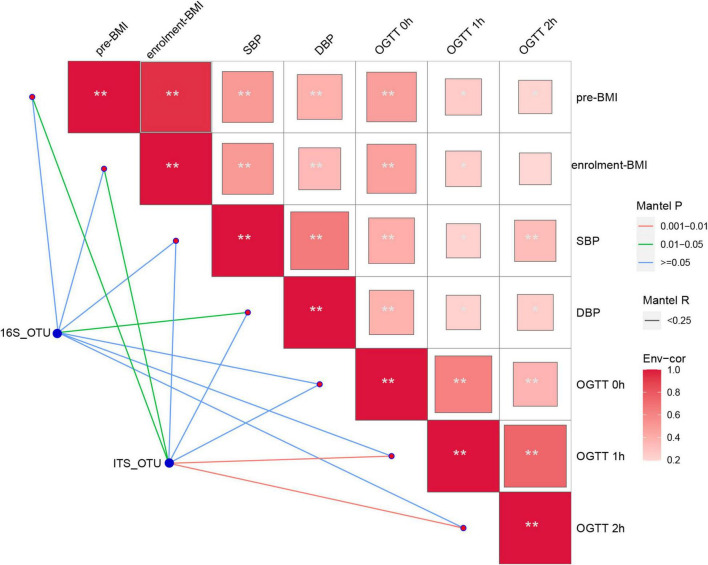
Fungal OTUs and the 16S OTUs associated with clinical metabolic parameters was determined by Mantel analysis. The co-occurrence network shows the correlations of the fungal and bacterial microbiota with the clinical metabolic parameters. The color of lines between nodes indicate significant (red line: positive correlations with *P* < 0.01 and green line: positive correlations with *P* < 0.05) or non-significant (blue line) correlations. Blue nodes: bacterial and fungal microbiota involved in GDM group; red nodes: the clinical metabolic parameters. Asterisk in square indicating relationship between the clinical metabolic parameters, **P < 0.01, *P < 0.05.

As reported, gut bacteria and fungi coexist and interact with each other, and yeast mannose is a viable food source for *Bacteroides thetaiotaomicron* (Cuskin et al., 2015). To confirm whether the metabolic disorder can be driven by bacteria or fungi during GDM in pregnancy, the bacterial abundance data were used from our previous study (Wu et al., 2021). Mantel test was performed. Figure 5 indicates that the gut mycobiome may be an important determinant of clinical metabolic parameters, with OGTT values (1 and 2 h) and BMI values being significantly associated with fungal OTUs, rather than the 16S data. Furthermore, we herein investigated the correlation by Spearman analysis and found that the abundances of some specific probiotic fungal taxa, namely, *Penicillium*, *Fusarium*, and *Kluyveromyces*, were negatively correlated with pathogenic bacteria, such as *Enterococcus*, suggesting the antipathogenic effects of *Penicillium*, *Fusarium*, and *Kluyveromyces* (Supplementary Figure 4).

## Discussion

Fungi play an important role in the intestinal tract, with dysbiosis contributing to obesity and diabetes (Borges et al., 2018; Zhang et al., 2021) and influencing health and disease. However, knowledge regarding the composition and function of the gut mycobiota in GDM patients, especially the influence of the diet on fungal distribution, is still limited. In this study, we comprehensively compared the gut mycobiome of GDM patients and healthy subjects using a culture-independent Illumina NovaSeq 6000 platform. The aim of the present study was to reveal the gut fungal signature in GDM subjects and their associated changes in GDM-W2 samples after diet intervention for 2 weeks. A marked shift was observed in the microbiota composition at the genus level in GDM patient samples compared with that of healthy samples, rather than at the phylum level. In addition, the fungal distribution of GDM-W2 samples with short-term dietary management and that of healthy samples across developmental stages (2 weeks without any intervention) were identified. Our findings highlight the potential significance of the gut mycobiome in gestation health and have implications for experimental metabolic studies in the gut microbiota field.

As expected, there was a mycobiome shift with GDM, as shown by the PCoA plot, with lower beta diversity and observed species in the Venn diagram. Abundances of several bacterial groups at the genus level were detected to differ in the GDM and healthy groups, and *Hanseniaspora*, *Torulaspora*, and *Candida* contributed to the variation in the GDM patient mycobiome. Among them, *Hanseniaspora* and *Torulaspora*, belonging to the phylum Ascomycota, were significantly more abundant in GDM patient samples at enrollment. A recent study showed that 6 weeks of a high-fat diet increased the abundances of various fungal taxa, including *Candida* and *Hanseniaspora* (van der Merwe et al., 2020). Of particular interest, we revealed that *Hanseniaspora* abundance was positively correlated with blood pressure and higher blood glucose in the OGTT test at 0 h in the GDM patient samples at enrollment. The relationships between *Hanseniaspora* abundance and glucose metabolism, such as with NAFLD (Demir et al., 2022), have recently been discovered, leading to a clue to this taxa’s role on host metabolic disorder. Members of *Candida* are known to be related to obesity. Obese subjects have been shown to present a higher prevalence of *C. albicans*, which also presented a positive correlation with weight gain and fat mass and showed a negative association with high-density lipoprotein levels and lean mass (Garcia-Gamboa et al., 2021). Another key gut commensal species assigned to the genus *Candida*, *C. parapsilosis*, was proven to foster diet-induced obesity, and the increase in free fatty acids in the gut due to the production of fungal lipases is confirmed as one mechanism to promote obesity (Sun et al., 2021a) and highlights the importance of therapeutic strategies targeting gut fungi. Abundance of another GDM-contributing genus, *Zygosaccharomyces*, exhibited a positive correlation with blood glucose levels at three time points, which was significantly reduced by 2 weeks of diet management.

Conversely, our data demonstrated that various beneficial fungal populations, *Penicillium*, *Ganoderma*, *Fusarium*, *Chaetomium*, and *Heterobasidion*, had significant effects on healthy patient clustering. In addition, the occurrences of *Penicillium*, *Ganoderma*, *Fusarium*, *Chaetomium*, *Heterobasidion*, *Kluyveromyces*, and *Wickerhamomyces* were observed to be negatively correlated with OGTT values, suggesting probiotic potential. *Penicillium*, one of the important gut commercial fungi, showed a significantly higher prevalence in healthy women than in GDM patients. The level of *Penicillium* has also been reported to be negatively associated with parameters of body fatness (Mar Rodriguez et al., 2015). A member of *Penicillium* showed potential inhibitory activity on pancreatic lipase (Gupta et al., 2015), which has been widely recognized as one of the safest drug targets for diet-induced anti-obesity development. *Penicillium* is also well documented to produce inhibitors of α-glucosidase (Malik et al., 2020), which is responsible for converting starch to mono-saccharides and is considered a key therapeutic target for the management of T2D. Of particular interest, we revealed anti-inflammatory effects of the probiotic fungus according to the negative correlation between abundances of the pathogen *Enterococcus* and *Penicillium*, *Fusarium*, *Kluyveromyces* (Supplementary Figure 4), which is consistent with the findings showing the antimicrobial efficacy of *Penicillium* (Rozman et al., 2017). *Penicillium* and *Fusarium* can also produce secondary bile acids which function in multiple metabolic processes and benefit for the host health (Kollerov et al., 2016).

Another important function of the gut probiotic mycobiome that is often credited for much of its health benefits is the production of polysaccharides. Understanding of the role of polysaccharides has evolved from their effects on modulating blood glucose levels to their anti-inflammatory activities, which are intimately related to multiple metabolic processes, and these findings implicate important functions of polysaccharides in metabolic homeostasis, such as obesity and type 2 diabetes. Polysaccharides exhibit strong anti-inflammatory activities by inhibiting the expression of proinflammatory cytokines, such as interleukin-6 (IL-6) and interleukin-1β (IL-1β) induced by LPS (Wen et al., 2022). Polysaccharides are the predominant bioactive components in *Ganoderma lucidum* spores, which decrease the blood glucose levels of HFD-induced diabetic mice (Shao et al., 2022). The production of *Ganoderma lucidum* was reported to reduce insulin resistance by activating AMPK signaling in obese mice (Lee et al., 2020). In this study, *Ganoderma*, the dominant genus in healthy subject samples, had a significant explanatory effect on the variation across healthy samples, and its abundance showed negative correlations with OGTT values at 0, 1, and 2 h (*P* < 0.05). The most important finding was that the level of *Ganoderma* markedly increased in GDM-W2 samples after 2 weeks of diet intervention, suggesting the beneficial effect of diet on GDM patient management through its modulation of the compositions of some probiotic fungi. Another polysaccharide-producing fungus was *Chaetomium*, members of which were found to have antibacterial activity (Sun et al., 2021b). Anti-inflammatory polysaccharides from *Chaetomium nigricolor* were reported to inhibit nuclear factor-kappa B and c-Jun N-terminal kinase activation, in turn suppressing levels of proinflammatory mediators and cytokines (Kim et al., 2020). In addition, another key effect of a polysaccharide produced by *Chaetomium* was its ability to reduce the body weight gain of mice (Shao et al., 2022). These findings may suggest a probiotic role for the polysaccharide-producing fungi *Ganoderma* and *Chaetomium* in the metabolic processes of healthy pregnancy through their fungal product, polysaccharides. Overall, this study provides potential insight for the promising application of diet intervention and polysaccharides as preservatives in the future.

Therefore, to further reveal the effort of diet intervention during GDM patient pregnancy, we analyzed the mycobiome pattern and compared differences between the GDM and GDM-W2 groups. Similar to the findings in our previous study, we found that the role of short-term diet management in GDM patient treatment is associated with the change in some specific fungi rather than a shift in the gut microbial pattern. The abundance of the probiotic genus *Ganoderma* was elevated in GDM-W2 samples after diet management, while the abundances of the GDM-related genera *Hanseniaspora*, *Zygosaccharomyces*, and *Armillaria* were depleted in GDM-W2 samples, suggesting the promising role of short-term diet intervention by modulating these gut fungi. Moreover, an increase in the abundances of phylum Ascomycota and the probiotic genera *Penicillium*, *Mortierella*, *Cutaneotrichosporon*, *Wickerhamomyces*, *Chaetomium*, *Fusarium*, *Kluyveromyces*, *Heterobasidion*, *Trametes*, and *Ganoderma* relative to those of healthy-W2 was observed. The findings indicate the beneficial change in fungal composition of healthy pregnant women across 2 weeks of developmental stages.

In summary, it is well suggested that diet contributes to the gut mycobiome composition in healthy mice (Mims et al., 2021; Shuai et al., 2022). Few studies have examined the gut mycobiome of GDM patients before and after diet invention. Uniquely, in the present study, the data indicate that the gut mycobiome in GDM subjects is highly variable and responds to dietary disturbances, providing a deeper understanding that GDM mycobiota will also help identify new targets for GDM prevention and treatment. Additionally, we highlighted some specific probiotic and fungus-derived products with plausible effects on host metabolism. Modulating the gut mycobiome, especially the abundance of polysaccharides-producing bacteria, via short-term diet intervention could be a promising strategy in the alternative treatment of metabolic disorders in GDM patients. Our study is limited by (1) the lack of long-term diet intervention to study the dynamic alteration of the GDM development; and (2) the lack of metagenomic sequencing data to reveal the metabolic pathways of the key taxa. Therefore, our suggestion of the prevalence of specific taxa with divergent metabolites calls for future metagenomic sequencing studies to reveal the metabolic pathways of the key fungal taxa in GDM mycobiota. Long-term observation may be more valuable to study the dynamic alteration of the GDM patient gut microbiota.

## Data Availability Statement

The sequence data in this study have been deposited in the GenBank Sequence Read Archive under BioProject accession number PRJNA813503.

## Ethics Statement

The studies involving human participants were reviewed and approved by the Conjoint Health Research Ethics Board of Peking University People’s Hospital. The patients/participants provided their written informed consent to participate in this study.

## Author Contributions

HM, QM, and NW: statistical analyses. NW and WY: sequencing analyses and management. PL, GL, and WY: project supervision and manuscript writing. All authors contributed to the article and approved the submitted version.

## Conflict of Interest

The authors declare that the research was conducted in the absence of any commercial or financial relationships that could be construed as a potential conflict of interest.

## Publisher’s Note

All claims expressed in this article are solely those of the authors and do not necessarily represent those of their affiliated organizations, or those of the publisher, the editors and the reviewers. Any product that may be evaluated in this article, or claim that may be made by its manufacturer, is not guaranteed or endorsed by the publisher.

## Footnotes

1
http://qiime.org/scripts/split_libraries_fastq.html

2
http://www.drive5.com/usearch/manual/uchime_algo.html
