# Supplementary material for: The Gut Mycobiome Characterization of Gestational Diabetes Mellitus and Its Association With Dietary Intervention
Source: Front Microbiol. 2022 Jun 15;13:892859. doi: 10.3389/fmicb.2022.892859 (PMC9240440; doi:10.3389/fmicb.2022.892859)
Supplement: Supplementary file 1 [file Data_Sheet_1.DOCX]

Supplementary Material

# Supplementary Figures

**Supplementary Figure 1.** Flow chart illustrating the recruitment strategy of GDM and healthy subjects.

**Supplementary Figure 2.** Comparison of the relative abundances at the species level among the GDM and healthy groups. Mann-Whitney test. ***P*<0.01.

**Supplementary Figure 3.** Heatmap analysis of the differential taxa of the top 35 genera in the four groups, including the GDM and healthy and the GDM-W2 and healthy-W2 groups.

**Supplementary Figure 4.** Microbial interaction patterns. The top 20 abundance of bacteria and top 20 fungi was analysed using Spearman test. ***P*<0.01, **P*<0.05.

# Supplementary Tables

**Supplementary table 1**

|  | R^2^ | *P* value |
| --- | --- | --- |
| Age | 0.15 | 0.03 |
| Pre-Prepregnancy BMI | 0.15 | 0.04 |
| Enrollment BMI | 0.12 | 0.06 |
| Systolic BP | 0.26 | 0.002 |
| Diastolic BP | 0.34 | 0.0005 |
| OGTT 0h | 0.22 | 0.05 |
| OGTT 1h | 0.38 | 0.0005 |
| OGTT 2h | 0.47 | 0.0005 |
