# Supplementary figures and images for: The Gut Mycobiome Characterization of Gestational Diabetes Mellitus and Its Association With Dietary Intervention
Source: Front Microbiol. 2022 Jun 15;13:892859. doi: 10.3389/fmicb.2022.892859 (PMC9240440; doi:10.3389/fmicb.2022.892859)

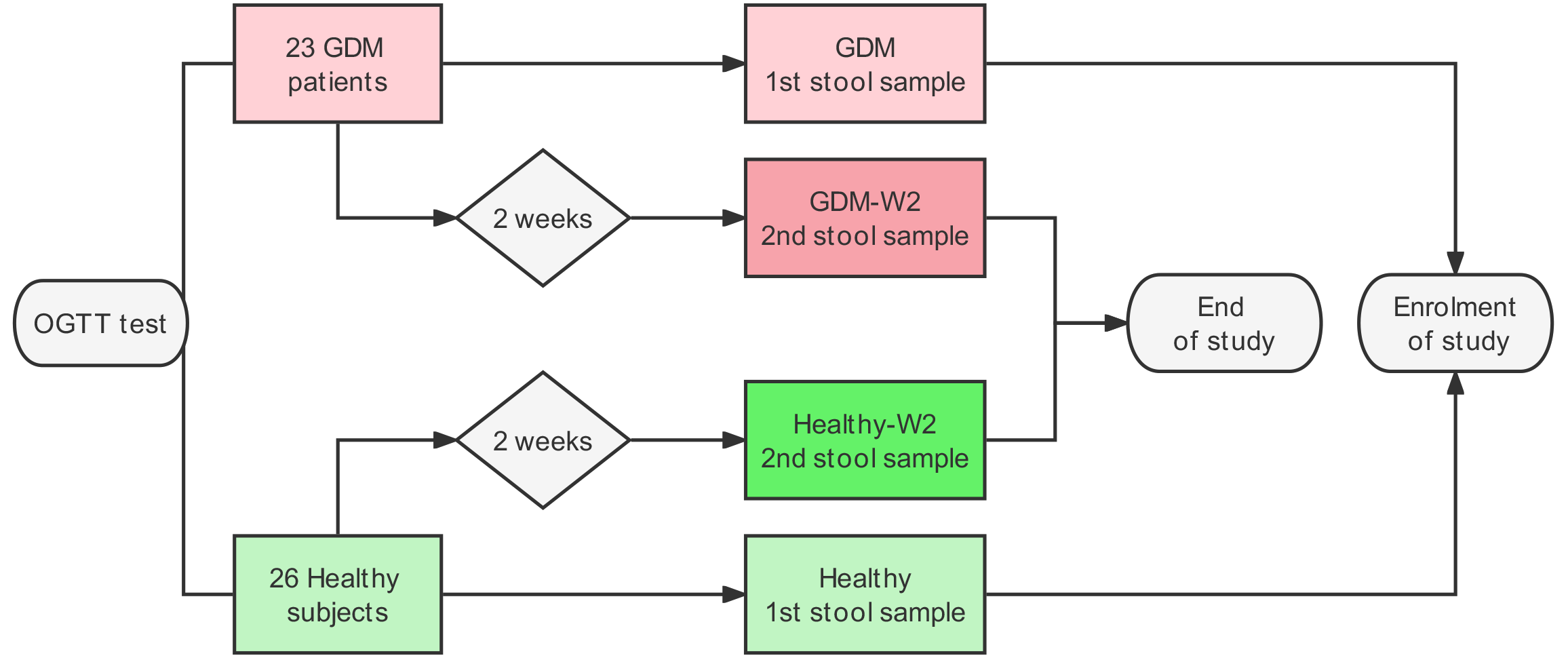

Supplement: Supplementary file 2 [file Image_1.tif]

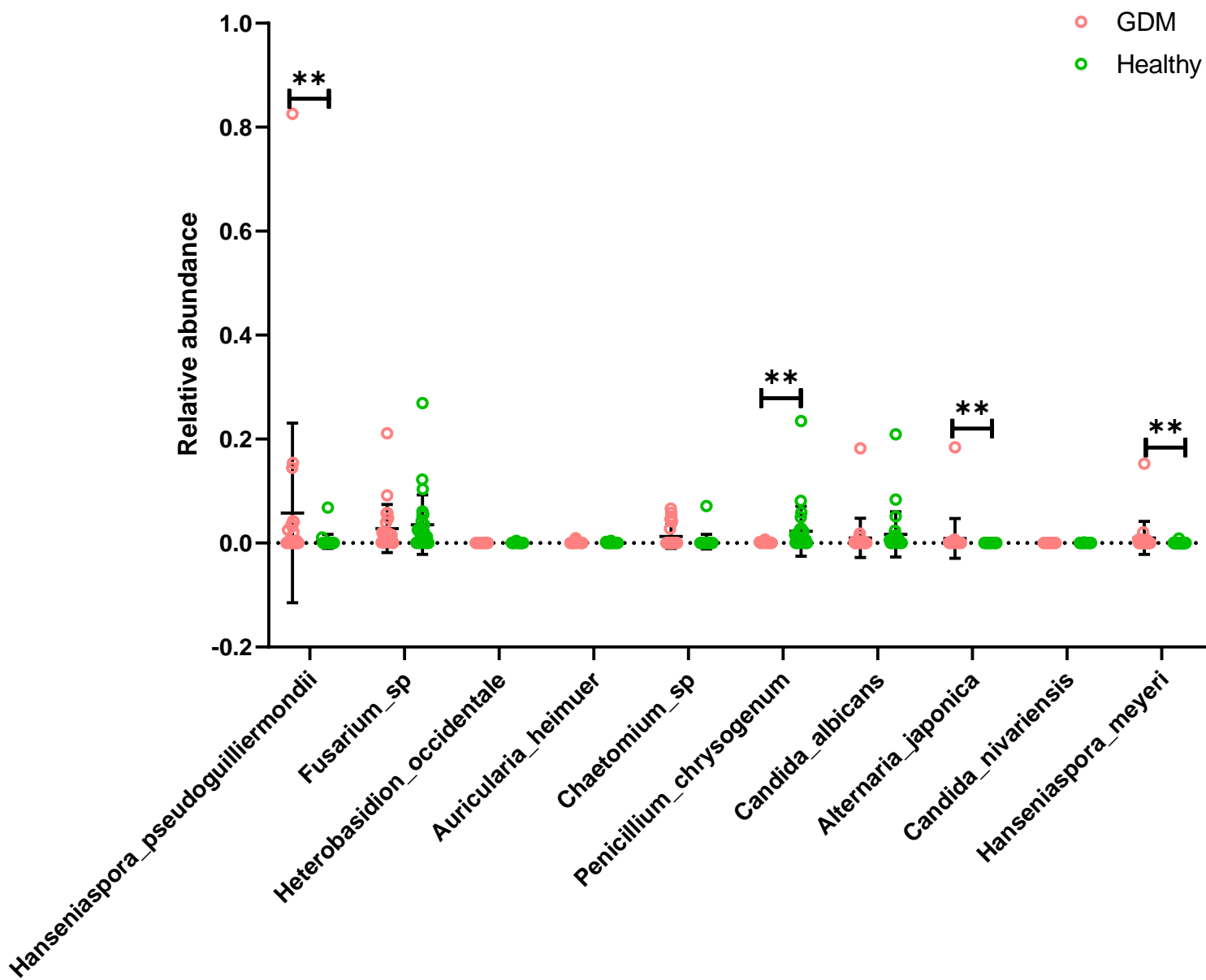

Supplement: Supplementary file 3 [file Image_2.PDF]

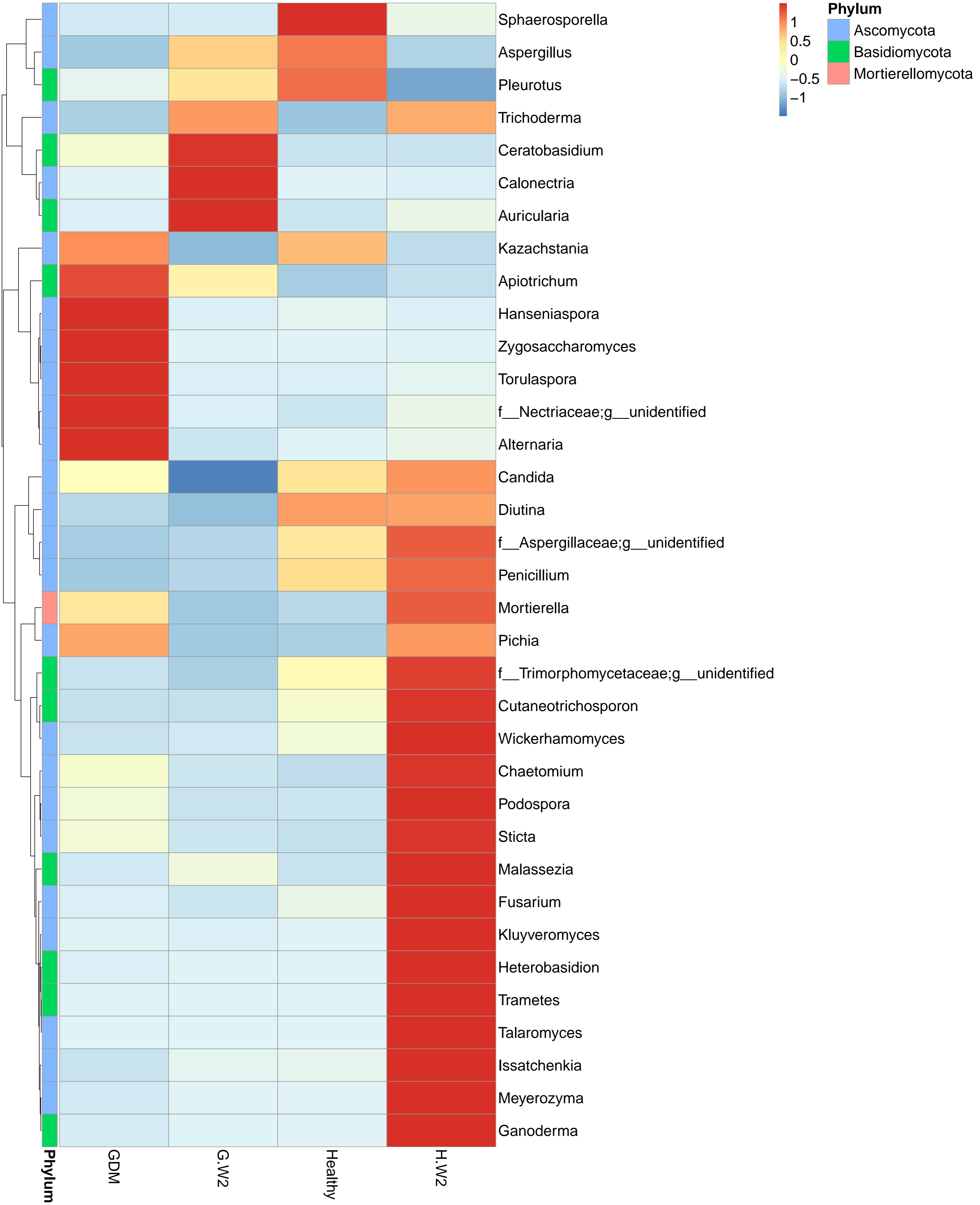

Supplement: Supplementary file 4 [file Image_3.PDF]

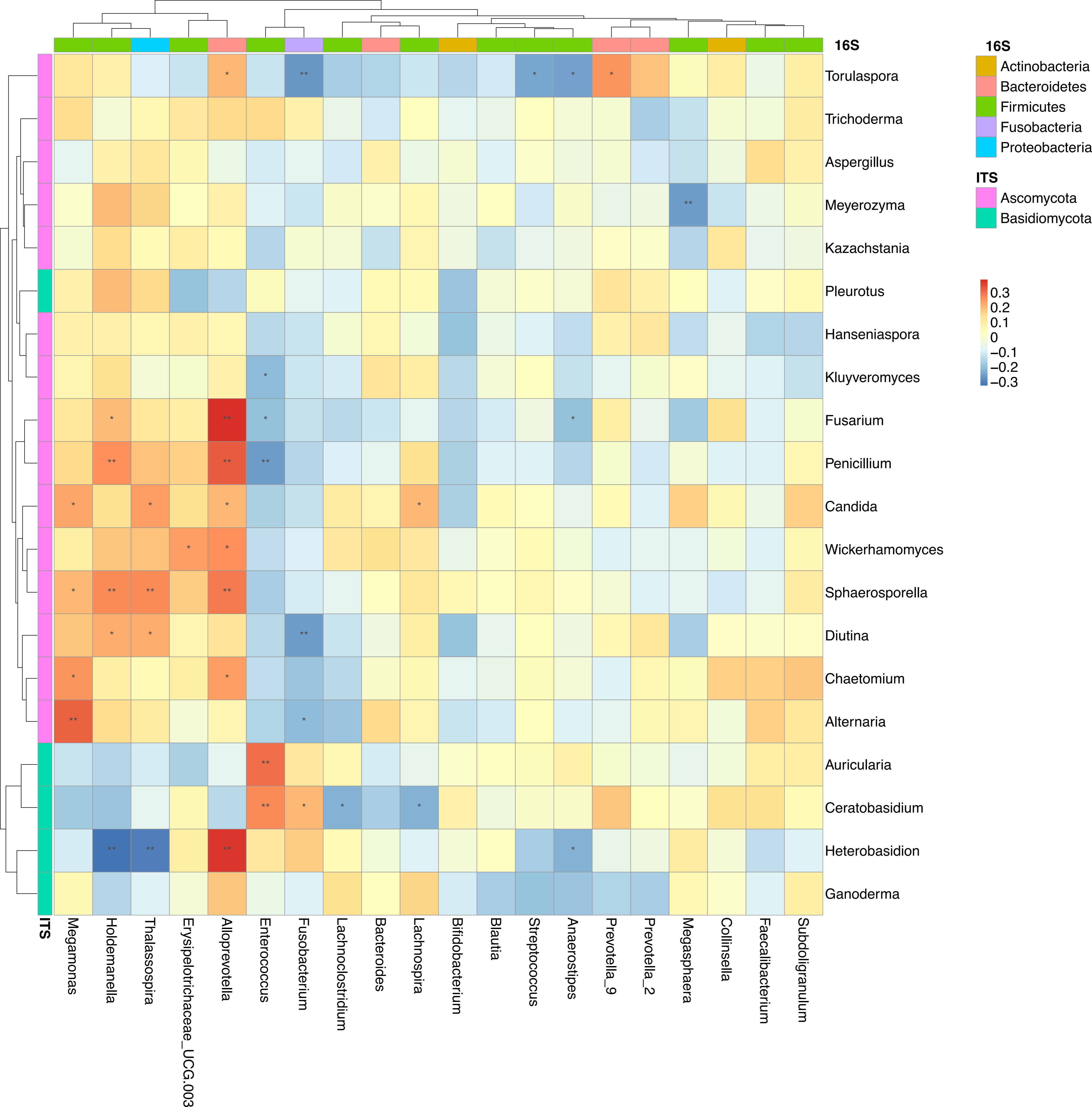

Supplement: Supplementary file 5 [file Image_4.PDF]
